# Phytochemical Composition of the Decoctions of Greek Edible Greens (Chórta) and Evaluation of Antioxidant and Cytotoxic Properties

**DOI:** 10.3390/molecules23071541

**Published:** 2018-06-26

**Authors:** Eleni V. Mikropoulou, Konstantina Vougogiannopoulou, Eleftherios Kalpoutzakis, Aimilia D. Sklirou, Zoi Skaperda, Joëlle Houriet, Jean-Luc Wolfender, Ioannis P. Trougakos, Dimitrios Kouretas, Maria Halabalaki, Sofia Mitakou

**Affiliations:** 1 Department of Pharmacognosy and Natural Products Chemistry, Faculty of Pharmacy, National and Kapodistrian University of Athens, Panepistimiopolis Zografou, 15771 Athens, Greece; elenamik@pharm.uoa.gr (E.V.M.); nadia_voug@pharm.uoa.gr (K.V.); elkalp@pharm.uoa.gr (E.K.); mitakou@pharm.uoa.gr (S.M.); 2 Department of Cell Biology and Biophysics, Faculty of Biology, National and Kapodistrian University of Athens, Panepistimiopolis Zografou, 15784 Athens, Greece; asklirou@biol.uoa.gr (A.D.S.); itrougakos@biol.uoa.gr (I.P.T.); 3 Department of Biochemistry and Biotechnology, University of Thessaly, 41221 Larissa, Greece; zoskaper@bio.uth.gr (Z.S.); dkouret@uth.gr (D.K.); 4 Phytochemistry and Bioactive Natural Products, School of Pharmaceutical Sciences, University of Geneva, University of Lausanne, CMU—Rue Michel-Servet 1, 1206 Geneva, Switzerland; Joelle.Houriet@unige.ch (J.H.); Jean-Luc.Wolfender@unige.ch (J.-L.W.)

**Keywords:** edible greens, chórta, Mediterranean diet, decoction

## Abstract

Wild or semi-wild edible greens (chórta) are an integral part of the traditional Greek Mediterranean diet due to their nutritional value, containing various phytonutrients beneficial to human health. Water-based decoctions of chórta are widely consumed in Greek alternative medicine as health promoting agents. This study examined the chemical profile of the decoctions of eight edible plants, *Cichorium intybus*, *C. endivia*, *C. spinosum*, *Crepis sancta*, *Sonchus asper*, *Carthamus lanatus*, *Centaurea raphanina*, and *Amaranthus blitum*, by UPLC-ESI-HRMS and HRMS/MS analysis, to determine possibly bioactive constituents. The profiles of the plants from the Asteraceae family are dominated by the presence of phenolic acids and flavonoid derivatives, whereas the *A. blitum* decoction is rich in triterpene saponins. Interestingly, the *Centaurea raphanina* decoction was found to be extremely rich in flavanones, particularly in the aglycone pinocembrin. Further phytochemical investigation and fractionation of this extract resulted in the isolation and identification of five compounds: phlorin (**1**), syringin (**2**), pinocembrin (**3**), pinocembroside (**4**), and pinocembrin-7-*O*-neohesperidoside (**5**). The extracts were also tested for their antioxidant and differential cytotoxic activity against tumor cells. *C. raphanina* was found to be differentially toxic against metastatic tumor cells. In conclusion, we found that Greek edible greens are a rich source of bioactive secondary metabolites and their consumption could contribute to the maintenance of overall health.

## 1. Introduction

Greek diet and dietary habits constitute a branch of the Mediterranean diet renowned for its beneficial effects on human health, having been proven to contribute to decreased rates of heart disease, cancer, and neurodegenerative diseases [1,2,3,4]. Wild and semi-domesticated greens (chórta) form an integral part of the Greek dietary regime [5,6,7] and references about their consumption and medicinal qualities can be found in ancient texts of Theophrastus, Pliny, and Dioscoride [8]. Certain edible greens possess a particularly high phenolic content and exhibit a strong antioxidant activity [9,10,11]. Moreover, chórta are a valuable nutritional source, being rich in fiber, vitamins, and minerals, such as iron and zinc [12,13,14].

Chórta are often cooked or eaten raw in a salad. Some species are also popular for their roots, fruits, or even their edible flowers. Cooked chórta are particularly popular in everyday Greek cuisine, with the daily national availability of wild greens being 20 g/person [10]. Moreover, the ancient practice of consuming the remaining water after cooking the plants is common throughout the Mediterranean, since the water is believed to improve gastrointestinal and liver health and to act as an overall detoxifying agent [15,16].

**Figure 1:**
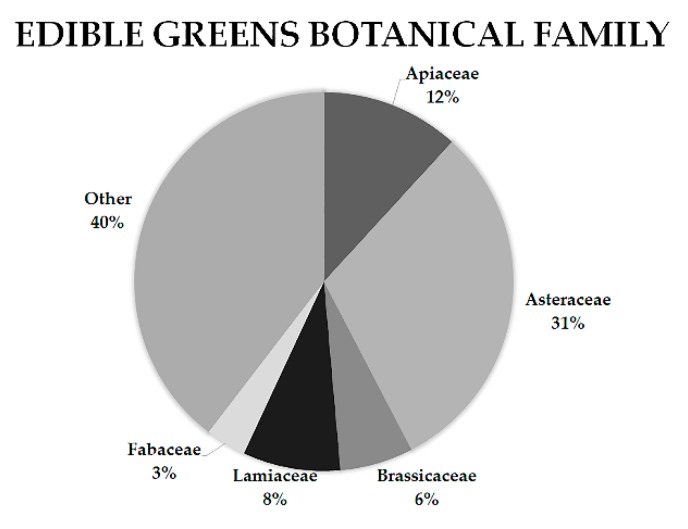
The botanical family of Mediterranean edible greens according to a survey conducted by the authors based on the available literature [5,6,12,13,17,18,19,20].

The term “chórta” is exclusively used in Greece to describe wild or semi-domesticated edible herbaceous plants that are consumed in the traditional Greek diet. Although ethnopharmacological interest in the consumption of the polyphenol-rich cooking water exists, phytochemical studies targeting the identification of bioactive natural products have been limited, as portrayed by the available literature. Most studies did not distinguish wild edible greens from aromatic plants, whereas other studies even included species known for their ripe fruits such as wild strawberries and figs in this category. According to the literature [5,6,12,13,17,18,19,20], most wild food plant species belong to the Asteraceae family (Figure 1), with the Cichoriae tribe being dominant. As expected, the more widespread the species, the more citations can be found about its consumption. Nevertheless, identifying and categorizing wild edible greens are difficult because each region uses their own common names to describe the plants. As a result, the same common name may be used to describe more than one species or one species may be found with various common names across. Furthermore, most greens are consumed before their flowering period, which adds to the complexity of their botanical identification in ethnopharmacological studies. Finally, even though many studies focused on the comparative analysis of wild edible greens, the studies examining plants from the Mediterranean region are still relatively few. Among these publications, the vast majority focused on the ethnopharmacological aspect of their consumption [19,21,22,23], whereas some were dedicated to the evaluation of certain biological activities of these plants [11,12]. Many articles analysed the phytochemical or pharmacological properties of specific genera or species; however, the comparative element is missing from the literature concerning the phytonutrients present in healthy diet regimes.

Based on our laboratory’s ongoing investigation of edible plants and their decoctions [24,25,26], this work characterises their phytochemical properties using dereplication methods for eight Greek edible greens using UPLC-Orbitrap MS. Driven by LC-HRMS analysis, targeted isolation was performed to purify and identify pinocembrin and its glycosylated derivatives, indicating an alternative and rich source of these nutrients in addition to propolis and honey. Furthermore, evaluation of the antioxidant and cytotoxic properties of these decoctions against tumor cells on several in vitro and cell-based models was performed.

## 2. Results

### 2.1. Preparation of Decoctions, Enrichement and Isolation

The first step in this work was to select the edible greens to be investigated and their procurement. The selection criteria were: the frequency of consumption in the Greek population, the availability, and the possibility of reliable botanical identification. To avoid misleading results, some greens were excluded from the initial list when the botanical characterization was not confirmed and/or multiple or confusing common names were used for a plant. Thus, *Centaurea raphanina* (agkinaráki), *Carthamus lanatus* (gkourounáki), *Cichorium endivia* (agourorádiko), *Cichorium intybus* (radíki), *Cichorium spinosum* (stamnagkáthi), *Crepis sancta* (ladáki), *Sonchus asper* (zochós), and *Amaranthus blitum* (vlíto) were subjected for this study. Following the common practice for their consumption, decoctions of each plant were prepared. These aqueous extracts represent the material consumed as a traditional remedy. Apart from the decoctions, enriched extracts were also prepared with the aid of the adsorption resin XAD7 HP, which has the ability to retain low molecular weight phenolic compounds while dismissing saccharides present in water extracts.

The treatment of all decoctions with XAD7 HP resulted in the elimination of sugars and the production of extracts enriched in small molecules that were forwarded to UPLC-HRMS-ESI(−) and HRMS/MS analysis for dereplication and identification of components. Specifically, in *Centaurea raphanina*, we observed that the water fraction containing the sugars after XAD treatment was rich in water soluble phenolic compounds that were not retained by the resin under the conditions used. Moreover, the published data related to its composition were rather limited, whereas no prior report exists for this decoction [27,28]. Therefore, we proceeded to perform the preparative separation of the crude decoction to isolate and identify molecules that were present both in the enriched and the crude decoction.

**Figure 2:**
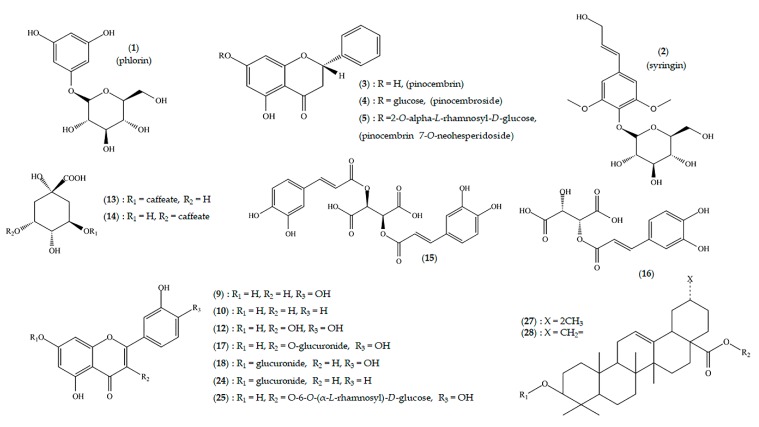
Chemical structures of the main secondary metabolites detected in chórta decoctions.

For the separation, Fast Centrifugal Partition Chromatography (FCPC) was employed after selecting the suitable biphasic solvent solution. Several systems were tested for their ability to equally partition the molecules contained in the extract between the two phases, and the final decision was made based on control by TLC. Three highly pure compounds were isolated in one step and two more were isolated after purification of the selected FCPC fractions with column chromatography. Specifically, phlorin (**1**), syringin (**2**), pinocembrin (**3**), pinocembroside (**4**), and pinocembrin-7-*O*-neohesperidoside (**5**) were obtained (Figure 2). Structure elucidation of compounds **1**–**5** was performed by means of 1D and 2D nuclear magnetic resonance (NMR), whereas all spectral data were in accordance with the literature [29,30,31,32]. NMR spectral data for pinocembroside (**4**) are presented in Figure S3 and Table S8.

Pinocembrin (**3**) is not a very common flavanone due to its structural motif with an unsubstituted B ring. Even if it is abundant in plants belonging to several plant families, pinocembrin is considered a characteristic component of honey and propolis, sometimes being one of the major metabolites. 

### 2.2. UPLC-HRMS-ESI(−) and HRMS/MS Profiling of the Enriched Chórta Decoctions

To gain insight into the composition of the decoctions or the enriched decoctions of *Centaurea raphanina* (agkinaráki), *Carthamus lanatus* (gkourounáki), *Cichorium endivia* (agourorádiko), *C. intybus* (radíki), *C. spinosum* (stamnagkáthi), *Crepis sancta* (ladáki), *Sonchus asper* (zochós), and *Amaranthus blitum* (vlíto), the extracts were subjected to UPLC-ESI(−)-Orbitrap HRMS analysis. Identification of the compounds was based on the interpretation of their HRMS and HRMS/MS spectra and on comparison with bibliographical references and online databases. The availability of accurate mass measurements (1–2 ppm) and the 30,000 mass resolution, or even higher in low mass regions, reaching 70,000 as assessed by the Orbitrap analyzer, enabled the prediction of the Elemental Compositions (EC) of the detected peaks with high confidence. Furthermore, the use of data-dependent methods based on the HRMS/MS spectra allowed an additional level of identification. Based on fragmentation patterns, we obtained characteristic MS/MS spectra. The error of all accurate mass measurements was lower than 5 ppm, and sometimes even less than 1 ppm.

In general, UPLC-ESI(−)-Orbitrap HRMS analysis of all extracts under investigation revealed the presence of flavonoids, flavonoid glycosides, and cinnamic and caffeoylquinic acid derivatives (Supplementary Material). Regarding the enriched decoction profile of *Centaurea raphanina*, the extract appeared to be rich in flavanone derivatives, and especially in pinocembrin (**3**) analogues (Table 1). The detection of pinocembrin derivatives in the *Centaurea* genus was previously reported [28]; however, more than six pinocembrin glycosides and diglycosides were detected in the extract in this case. Among them, pinocembrin arabinosyl glucoside (**6**) (*m/z* 549.1609, C_26_H_29_O_13_, RDBeq. 12.5) and pinocembrin neohesperidoside (**5**) (*m/z* 563.1763, C_27_H_31_O_13_, RDBeq. 12.5) and their acetylated forms (**7**, **8**) (*m/z* 591.1711 and *m/z* 605.1867, respectively), appeared to dominate the extract. In order to estimate the pinocembrin derivative levels in the extract, an HPLC-PDA-based relative quantitation was performed (Figure S4, Table S9, Supplementary Material). The results indicated that that pinocembrin arabinosyl glucoside, pinocembroside, and pinocembrin neohesperidoside are the major constituents in *Centaurea raphanina.*

**Figure 3:**
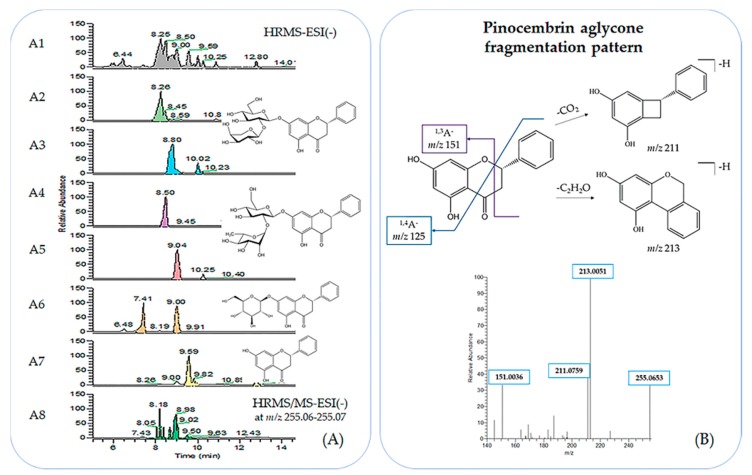
(**A**) A1: UPLC-ESI(−)-HRMS profile of *Centaurea raphanina* enriched decoction; A2: Extraction Ion Chromatogram (XIC) at *m/z* 549.16–549.17, pinocembrin arabinosyl glucoside (**6**); A3.:XIC at *m/z* 591.17–591.18, pinocembrin acetyl arabinosyl glucoside (**7**); A4: XIC at *m/z* 563.17–563.18, pinocembrin neohesperidoside (**5**); A5: XIC at *m/z* 605.18–605.19, pinocembrin acetyl neohesperidoside (**8**); A6: XIC at *m/z* 417.11–417.13, pinocembroside (**4**); A7: XIC at *m/z* 255.06–255.07, pinocembrin (**3**); and A8: HRMS/MS at *m/z* 255.06–255.07; (**B**) Pinocembrin (**3**) HRMS^2^ fragmentation pattern [35].

The pseudomolecular ion of pinocembrin (**3**) ([M − H]^−^ at *m/z* 255.0664 C_15_H_11_O_4_, RDBeq. 10.5) is a characteristic fragment ion that could be used for the detection of all pinocembrin-containing compounds through HRMS/MS spectra. As shown in Figure 3, the Extracted Ion Chromatogram (XIC) at *m/z* 255.06–255.07 of HRMS/MS singles out potential pinocembrin-related compounds. The identification of the flavonoid aglycone as the flavanone pinocembrin was possible by interpreting the molecule’s fragmentation pattern in its HRMS/MS spectrum. The results coincide with previous bibliographical references [33,34] and the identification of specific fragments is possible through the elemental composition provided by the High Resolution Orbitrap Analyzer and interpretation of these findings based on previous works on other flavanones’ fragmentation patterns [35] (Figure 3).

Finally, the extract’s profile was complemented by the presence of phenolic acids, flavonoid glycosides of the aglycones luteolin (**9**) (*m/z* 285.0406, C_15_H_9_O_6_, RDBeq. 11.5), apigenin (**10**) (*m/z* 269.0452, C_15_H_9_O_5_, RDBeq. 11.5) derivatives, and phenylpropanoids, such as 9-*O*-methylconiferin (**11**) (*m/z* 355.1396, C_17_H_23_O_8_, RDBeq. 6.6). Phlorin (**1**) and syringin (**2**) were also detected in the enriched extract of *C. raphanina*, as minor components, as their presence was more pronounced in the total water decoction.

The enriched decoction of *Carthamus lanatus* mainly included flavonoid derivatives, and more specifically, quercetin (**12**) and luteolin (**9**). The detection of fragment ions at *m/z* 301.0351 and *m/z* 285.0406, in the HRMS/MS spectrum that correspond to their pseudomolecular ions, indicated the presence of such derivatives [36]. Moreover, rather high levels of caffeoylquinic acid (CQA) isomers (*m/z* 353.0870, C_16_H_17_O_9_, RDBeq. 8.5) and dicaffeoylquinic acid isomers (*m/z* 515.1188, C_25_H_23_O_12_, RDBeq. 14.5) were detected in the extract. The distinction between the different isomers of these compounds was possible by interpreting their HRMS/MS spectra and the relative intensity of certain fragment ions. In particular, those of *m/z* 335.0755 [CQA − H_2_O − H^+^], quinate (*m/z* 191.0555), caffeate (*m/z* 179.0344), and *m/z* 173.0450 [quinic acid − H_2_O − H^+^]^−^ are characteristic for each isomer [37,38,39]. 

The *Cichorium endivia*, *C. intybus* and *Crepis sancta* extracts displayed an almost identical chemical profile, as they are all rich in phenolic acids, such as 3-caffeoylquinic (**13**) (*m/z* 353.0870), 5-caffeoylquinic (**14**) (*m/z* 353.0870), and mainly in two caffeoyltartaric acid derivatives: cichoric acid (**15**) (*m/z* 473.0723, C_22_H_17_O_12_, RDBeq. 14.5) and caftaric acid (**16**) (*m/z* 311.0405, C_13_H_11_O_9_, RDBeq. 8.5) [40]. Flavonoid derivatives were also present in the extract, with quercetin and luteolin glucuronides (**17**, **18**) (*m/z* 477.0670, C_21_H_17_O_13_, RDBeq. 13.5 and *m/z* 461.0721, C_21_H_17_O_12_, RDBeq.13.5, respectively) being the most prominent. The *Cichorium spinosum* decoction presented a somewhat similar profile, although it appears to be richer in cichoric acid (**15**) (*m/z* 473.0723) and possesses a greater chemical diversity in terms of the different cinnamic acid derivatives. Additionally, most flavonoids, derivatives of quercetin and luteolin, exist in their glucurinated form. A certain amount of sesquiterpene lactones, such as 8-deacetylmatricarin-8-*O*-sulphate [24] (**19**) (*m/z* 341.0695, C_15_H_17_O_7_S, RDBeq. 7.5) and lactupicrin (**20**) (*m/z* 409.1285, C_23_H_21_O_7_, RDBeq. 12.5), were detected in the decoction. The *Sonchus asper* decoction, although poorer in terms of chemical diversity, had significantly higher levels of small organic acids compared with the other extracts of the Cichoriae tribe, such as tartaric (**21**) (*m/z* 149.0095, C_4_H_5_O_6_, RDBeq. 2.5), quinic (**22**) (*m/z* 191.0562, C_7_H_11_O_6_, RDBeq. 2.5), and malic acid (**23**) (*m/z* 133.0147, C_4_H_5_O_5_, RDBeq. 2.5). Cinnamic acid derivatives compliment the profile (again cichoric acid (**15**) and caffeoylquinic acid isomers were the most prominent), along with glucurinated forms of the flavonoids luteolin (**18**) and apigenin (**24**) (*m/z* 461.0720 and *m/z* 445.0771, respectively).

Conversely, the *Amaranthus blitum* decoction was poor in phenolic substances, with only two flavonoid derivatives detected in relatively small amounts: rutin (**25**) (*m/z* 609.1448, C_27_H_29_O_16_, RDBeq. 13.5) and luteolin diglycoside (**26**) (*m/z* 593.1497, C_27_H_29_O_15_, RDBeq. 13.5). However, its chemical profile was dominated by the presence of the amphiphilic triterpene saponins that are the characteristic secondary metabolites of the genus *Amaranthus*. Based on existing studies [41], we estimated that all saponins detected in the extract (*m/z* 909.4083, C_45_H_65_O_19_, RDBeq. 13.5; *m/z* 955.4507, C_47_H_71_O_20_, RDBeq. 12.5; *m/z* 925.4404, C_46_H_69_O_19_, RDBeq. 12.5; and *m/z* 921.4445, C_47_H_69_O_18_, RDBeq. 13.5) were esters of either 2β,3β-dihyudroxyolean-12-en-28-oic acid (**27**) or 2β,3β-dihydroxy-30-norolean-12,20(29)-dien-28-oic acid (**28**). 

### 2.3. Evaluation of Cytotoxicity and Antioxidant Activity

**Figure 4:**
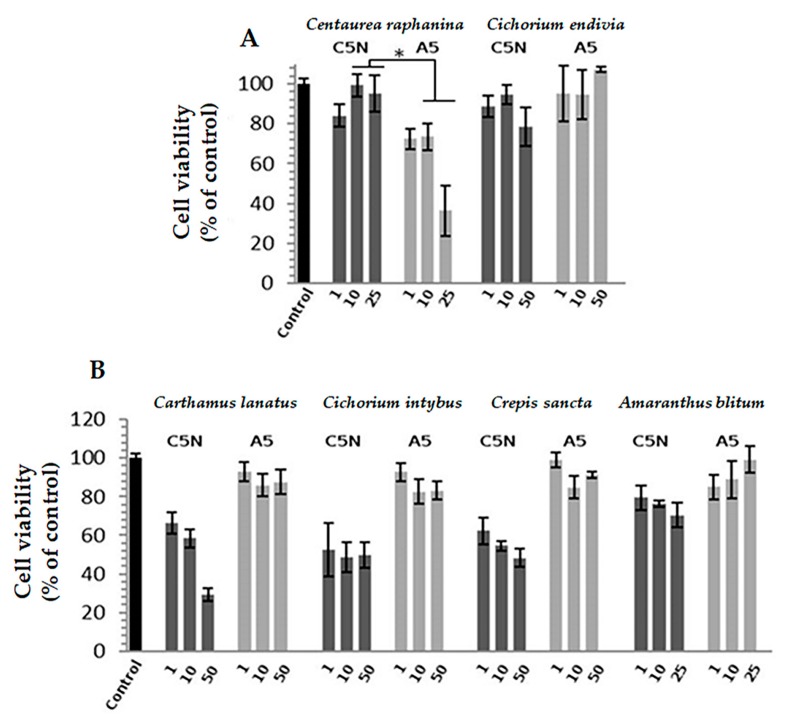
Selected water decoctions of edible chórta and differential toxicity against cancer cells. Comparative toxicities as determined by MTT assay of (**A**) *Centaurea raphanina* and *Cichorium endivia* and (**B**) *Carthamus lanatus*, *Cichorium intybus*, *Crepis sancta*, and *Amaranthus blitum* on C5N and A5 cells following incubation with the indicated concentrations (μg/mL) for 72 h.

As a second step, we examined the effects of the water decoctions on the viability of C5N and A5 cells by using the MTT assay. C5N and A5 cells were established following the mouse skin carcinogenesis protocol. C5N cells represent an immortalized highly differentiated non-tumorigenic cell line, whereas the A5 cell line represents metastatic spindle carcinoma [42,43]. Thus, these cell lines provide an excellent model for the investigation of differential cytotoxicity among non-cancerous and highly metastatic cells. The decoction of *Centaurea raphanina* was found to be differentially toxic against cancer metastatic A5 cells (Figure 4). Moreover, the enriched decoction of *Cichorium endivia* at a concentration of 1 μg/mL (Figure S2) appeared to be differentially cytotoxic to cancer cells compared to the immortalized non-tumorigenic cells. The water decoctions of *Carthamus lanatus*, *Cichorium intybus*, *Crepis sancta*, and *Amaranthus blitum* were found to be cytotoxic at the concentrations used, mostly in C5N cells.

As a third step, we evaluated the antioxidant activity of the enriched decoctions of the extracts and correlated the results with the chemical profile and cytotoxicity. According to the DPPH assay results, all the tested enriched decoctions exhibited strong free radical scavenging activity at low concentrations, except for *C. raphanina*. The half maximal inhibitory concentration (IC_50_) values ranged from 7.51 to 120.60 μg/mL. The extract exerting the highest antioxidant potency was *Cichorium endivia*, with an IC_50_ of 7.51 μg/mL (Table 2).

## 3. Discussion

Wild edible greens are inextricably linked to the traditional Greek Mediterranean diet [8,10], and their health benefits have been well studied and are renowned among health professionals and the general public [44,45]. Even in times of famine, wild edible greens have been a nutritional source for the Greek population and the knowledge concerning their collection, preparation, and their medicinal properties has been passed from generation to generation. During the post-II World War decades and following the arrival of new dietary trends and product commercialization, the usage of wild edible greens was neglected as they were characterized as “food of the poor” [46]; only to return to the diets of the Greek population as a valuable “superfood”. Several studies have studied specific genera or species that fall into the general category of edible greens; however, only a handful of attempts have been made to approach the issue in a broader context. This work emphasizes on the phytochemical analysis of the decoctions of eight edible greens and evaluates their antioxidant activity and differential toxicity against metastatic tumor cells.

According to our findings, the plants belonging to the Asteraceae family are rich sources of phenolic compounds and specifically of phenolic acids and flavonoid glycosides. In particular, the plants belonging to the Cichoriae tribe (*Cichorium spp*., *Crepis sancta*, *Sonchus asper*) presented a somewhat similar profile, with caffeoyltartaric acid derivatives, such as cichoric and (**15**) caftaric acid (**16**), being the most prominent secondary metabolites of the extracts, followed by luteolin derivatives and hydroxycinnamates, such as caffeoylquinic acid isomers. Cichoric acid (**15**) has received attention in the scientific community due to its numerous biological activities, including anti-diabetic and anti-inflammatory activities [47,48,49,50].

*Carthamus lanatus*’ decoction presented a simpler profile than the other extracts. Even though the decoction mainly contained luteolin and apigenin derivatives, as well as caffeoylquinic and dicaffeoylquinic acid isomers, it appears to possess one of the highest antioxidant potencies among the extracts. Notably, the *Amaranthus blitum* decoction, even though poor in phenolic compounds, contained high levels of triterpene saponins, the presence of which is expected in plants of the Amaranthaceae family [51]. Finally, the absence of betacyanins in the extract is noteworthy: although they constitute a classic biomarker of the *Amaranthus* genus, they were found in very small amounts in the species [52] but they are also extremely susceptible to degradation by heating and exposure to radiation [53].

Notably among all chórta examined in this work, the most interesting chemical profile was attributed to the decoction of *Centaurea raphanina* due to the predominant presence of pinocembrin analogues. Pinocembrin (**3**) is a constituent of a variety of well-studied source materials, such as propolis and licorice, that have been long used in traditional medicine for their antibacterial and anti-inflammatory activities. Pinocembrin and its glycosides have exhibited anthelminthic, anti-inflammatory, cardioprotective, neuro-protective, and anti-tumor properties in in vitro and in vivo models [54,55,56,57,58,59,60,61,62]. In accordance, we found that the decoction of *Centaurea raphanina* was differentially toxic against cancer cells. Yet, given the frequent consumption of chórta, and as other decoctions were found to mostly affect differentiated cells, more studies should be performed, ideally at the single molecule level, to better understand the impact of these decoctions in mammalian cells. Interestingly, the enriched decoction of *C. raphanina*, which accounted for the majority of the phenolic constituents of the crude decoction tested for cytotoxicity, showed low antioxidant potency in the DPPH assay. This property is likely related to its differential toxicity against tumor cells as the increased anti-oxidant activity enhances the growth of established tumors [63]. This is partially due to the lack of hydroxyl groups in the pinocembrin B-ring compared with the flavanols and flavones present in the other decoctions that showed high antioxidant potential. 

## 4. Materials and Methods

### 4.1. Materials and Chemicals

For the preparation of the decoctions, distilled water was used. LC-MS grade solvents were used for the phytochemical analysis of the extracts: Acetonitrile (Carlo Erba Reagents, Val de Reuil, France), Formic Acid Optima^TM^ (Fisher Chemical, Pittsburg, PA, USA), Methanol LiChrosolv^®^ (Merck, Burlington, MA, USA), and Ultrapure water from a Direct-Q^®^ Water Purification System (Merck). For the fractionation of the *Centaurea raphanina*’s extract, analytical grade solvents (*n*-butanol, ethyl acetate, dichloromethane, and methanol) were employed.

### 4.2. Plant Material Extraction, Enrichment of Extracts and Isolation of Principal Components

In total, eight chórta samples were purchased from local markets in Athens, Greece in the spring of 2015: *Centaurea raphanina* (agkinaráki), *Carthamus lanatus* (gkourounáki), *Cichorium endivia* (agourorádiko), *C. intybus* (radíki), *Crepis sancta* (ladáki), and *Sonchus asper* (zochós), and two were purchased in July of 2016: *Cichorium spinosum* (stamnagkáthi), *Amaranthus blitum* (vlíto). Seven samples belonged to the Asteraceae family and one belonged to the Amaranthaceae family. The Greek common names are provided in parentheses. The samples were immediately transferred to the Laboratory of Pharmacognosy and Natural Products Chemistry and botanically characterized. A sample specimen can be found in the herbarium of the laboratory. The fresh clean young leaves and stems were cooked in the traditional method, by boiling with water in an analogy of 500 g of plant material/1 L of water, for approximately 20 min. The decoctions were left to cool at room temperature, then filtered through paper and evaporated to dryness, first with a rotary evaporator (Buchi, Flawil, Switzerland) at 40 °C and then by freeze-drying. Five of the extracts: *Centaurea raphanina*, *Carthamus lanatus*, *Crepis sancta*, *Cichorium endivia*, and *Cichorium intybus*, were enriched using XAD7 HP Amberlite^®^ adsorption resin. For this process, the extract was first diluted in water and the activated resin was added. The solution was stirred for a few hours so that the medium polarity compounds were bound to the resin’s surface. The solution was then filtered through filter paper, and alcohol was added to the residue and the solution was stirred for a few hours. The compounds of interest were released from the resin to the organic solvent, which was then evaporated to dryness using a rotary evaporator (Buchi). All of the dry extracts were subjected to UPLC-HRMS analysis.

For the isolation of key components from the extracts of *Centaurea raphanina*, Countercurrent Partition Chromatography was mainly employed. An FCPC system with a 200 mL rotor (Rousselet-Robatel, Annonay, France) was used, combined with a Lab Alliance preparative pump and a Buchi B684 fraction collector. For the separation of the *Centaurea raphanina* decoction (0.91 g), the chosen biphasic system (n-butanol/ethyl acetate/water 4:1:5, 3 L) was prepared prior to analysis in a 5 L separatory funnel, where the two phases were left to separate. The rotor was filled with the lower stationary phase with a flow rate of 10 mL/min, and the system was equilibrated with the passing of the upper mobile phase with a flow rate of 7 mL/min and the rotation set to 1000 rpm. The retention volume of the stationary phase was 112 mL. In total, 121 fractions of 10 mL each were collected in ascending mode, whereas an additional 19 fractions were collected in extrusion mode. After TLC inspection, 11 joined fractions were obtained (A–K). Phlorin (**1**) (58.2 mg) and syringin (**2**) (15.3 mg) were isolated in one step from the initial FCPC separation (fractions D and F, respectively). Fraction B (113.5 mg) included the pinocembrin (**3**) analogues and was subjected to column chromatography with the use of normal phase silica gel (dichloromethane/methanol, step gradient elution 100:0, 98:2, 95:5, 90:10, 85:15, 80:20, 50:50) for the isolation of pinocembrin (**3**) (5.2 mg), pinocembrin 7-*O*-glucoside (**4**) (8.1 mg), and pinocembrin-7-*O*-neohesperidoside (**5**) (12.1 mg). 

### 4.3. Qualitative Composition Analysis of chórta Extracts: UPLC-ESI(−)-HRMS and HRMS/MS Conditions

Liquid chromatography analysis for the extracts was performed on an Acquity^®^ UPLC System (Waters, Milford, MA, USA). For all extracts, detection was performed on an LTQ-Orbitrap^®^ XL hybrid mass spectrometer equipped with an ESI source (Thermo Scientific, Waltham, MA, USA). For qualitative analyses, separation was achieved on an Ascentis^®^ C18 column (150 × 2.1 mm, 3 μm, Supelco Analytical, Bellefonte, PA, USA) using a water gradient containing 0.1% (*v*/*v*) formic acid (A) and acetonitrile (B). Elution started at 95% A, which decreased to 5% A in 23 min. These conditions were maintained for 3 min before returning to initial conditions in 2 min for a 3-min re-equilibration (31 min in total). The column was maintained at 40 °C and the flow rate was set to 0.4 mL/min. Water extracts (10 μL at 250 μg/mL) were injected. HRMS data were acquired in negative mode in the full scan *m/z* range of 95–1000 with a resolution of 30,000. Data-dependent acquisition was simultaneously performed using a CID value of 35% and a mass resolution of 7500. Capillary temperature was set to 350 °C and the source voltage was 2.7 kV. Tube lens and capillary voltage were respectively tuned at −100 V and −30 V. Nitrogen was used as the sheath gas (40 arbitrary units) and auxiliary gas (10 arbitrary units). Spectral interpretation was performed using the Xcalibur^TM^ (Version 2.2, Thermo Scientific) software.

### 4.4. DPPH (2,2-diphenyl-1-picrylhydrazyl) Radical Scavenging Assay

Free-radical scavenging capacity of the *C. spinosum*, *C. intybus*, *C. sancta*, *C. endivia*, and *S. asper* extracts were evaluated using the DPPH radical. The remaining extracts were not tested due to poor solubility. Briefly, a 1.0 mL freshly-made methanolic solution of DPPH radical (100 μΜ) was mixed with tested extract solution at different concentrations (0.5–100 μg/mL). The contents were vigorously mixed, incubated at room temperature in the dark for 20 min, and the absorbance was measured at 517 nm. The measurement was conducted on a Hitachi U-1900 radio beam spectrophotometer (Tokyo, Japan). In each experiment, the tested sample alone in methanol was used as blank and DPPH alone in methanol was used as control.

The percentage of radical scavenging capacity (RSC) of the tested extracts was calculated according to the following equation:

RSC (%) = [(A_control_ − A_sample_)/A_control_] × 100

where A_control_ and A_sample_ are the absorbance values of the control and the test sample, respectively. Moreover, in order to compare the radical scavenging efficiency of the extracts, the IC_50_ value showing the concentration that caused 50% scavenging of DPPH radical was calculated from the graph plotting RSC percentage against extract concentration. All experiments were performed in triplicate on at least two separate occasions.

### 4.5. Cell Lines and Cell Culture Conditions

C5N immortalized keratinocytes and A5 aggressive spindle cancer cells were a kind gift provided by Dr. Zoumpourlis (National Hellenic Research Foundation, Athens, Greece) [42]. Cells were cultured in Dulbecco’s modified Eagle’s medium (Thermo Scientific), supplemented with 10% (*v*/*v*) fetal bovine serum and 2 mM glutamine in a humidified incubator at 5% CO_2_ and 37 °C. In all experimental procedures, applied cells were subcultured when confluent by using a trypsin/EDTA solution (Thermo Scientific).

### 4.6. Cell Survival Assay

Cells were plated in flat-bottomed 96-well microplates. After 24 h, they were treated with different concentrations of the extracts for 72 h. Following the completion of the treatment, the medium was replaced by 3-(4,5-dimethylthiazol-2-yl)-2,5-diphenyltetrazolium bromide (MTT) dissolved at a final concentration of 1 mg/mL in serum-free, phenol red-free medium. The reduction of the dye by the living cells was allowed to occur for 3–4 h. The MTT solution was discarded and isopropanol was added to dissolve the formazan crystals. The absorbance of the solution was measured at a wavelength of 570 nm. Survival of the control cells was arbitrarily set to 100%. MTT assay was performed in triplicate. For statistical analysis, MS Excel was used. Statistical significance was evaluated using T-TEST. Data points correspond to the mean of the independent experiments and error bars denote standard deviation (SD); significance at *p* < 0.05 is indicated in graphs by a single asterisk.

## 5. Conclusions

Nutritional regimes from Greece are an important branch of the Mediterranean diet, known for its various beneficial health effects. Traditional medicine and chemoprevention knowledge connected with food consumption are being investigated as biological assays and chemical profiling techniques advance, resulting in the gradual elucidation of the role of phytonutrients. In this work, we attempted to clarify and provide insight into the composition of eight semi-wild greens from various families (*Cichorium intybus*, *C. endivia*, *C. spinosum*, *Crepis sancta*, *Sonchus asper*, *Carthamus lanatus*, *Centaurea raphanina*, and *Amaranthus blitum*), traditionally and regularly eaten in Greece. More importantly, we investigated the phytonutrients present in the respective decoctions that are traditionally consumed as health protecting agents. The investigated decoctions, amongst which *Centaurea raphanina* was unique, were found to be rich in pinocembrin analogues and were correlated with cytotoxic and antioxidant properties. This work contributes to the investigations into the beneficial health properties of the Mediterranean diet and the chemical elucidation of edible plants.

## Figures and Tables

**Table 1:** Retention time (Rt), HRMS data, and proposed identification of detected features in *Centaurea raphanina* enriched water decoctions by UHPLC-ESI(−)-HRMS.

Rt (min)	Detected *m/z* ([M − H]^−^)	HRMS/MS Fragment Ions (Relative Intensity)	Elemental Composition	RDBeq.	Δ (ppm)	Compound ^1^	Chemical Class
0.76	165.0408	-	C_5_H_9_O_6_	1.5	2.355	heptonic acid	organic acids
0.79	191.0564	-	C_7_H_11_O_6_	2.5	1.249	quinic acid	organic acids
0.90	133.0147	115 (100)	C_4_H_5_O_5_	2.5	3.559	malic acid	organic acids
0.96	191.0199	111 (100), 173 (16)	C_6_H_7_O_7_	3.5	1.121	citric acid	organic acids
0.99	287.0773	-	C_12_H_15_O_8_	5.5	4.201	phlorin	phloroglucinol
1.01	147.0304	129 (100), 85 (90)	C_5_H_7_O_5_	2.5	3.288	hydroxyglutaric acid	organic acids
3.81	353.0872	191 (100), 179 (7)	C_16_H_17_O_9_	8.5	−1.743	5-caffeoylquinic acid	hydroxycinnamates
3.83	417.1394	-	C_18_H_25_O_11_	6.5	−2.001	syringin formate	phenylpropanoids
4.40	179.0349	135 (100),	C_9_H_7_O_4_	6.5	−0.235	caffeic acid	hydroxycinnamates
4.64	337.0921	191 (100), 173 (12), 163 (8)	C_16_H_17_O_8_	8.5	−2.257	5-*p*-coumaroylquinic acid	hydroxycinnamates
4.74	431.1913	-	C_20_H_31_O_10_	5.5	−2.134	unknown	-
5.10	479.0822	317 (100)	C_21_H_19_O_13_	12.5	−1.886	myricetin glucoside	flavonol glycosides
5.83	463.0876	301 (100)	C_21_H_19_O_12_	12.5	−1.402	quercetin glucoside	flavone glycosides
5.88	447.0928	285 (100)	C_21_H_19_O_11_	12.5	−1.017	luteolin glucoside	flavone glycosides
5.91	461.0721	285 (100)	C_21_H_17_O_12_	13.5	−1.017	luteolin glucuronide	flavone glycosides
6.01	579.1346	285 (100)	C_26_H_27_O_15_	13.5	−1.560	luteolin pentoside hexoside	flavone glycosides
6.25	515.1188	353 (100), 299 (7)	C_25_H_23_O_12_	14.5	−1.416	4,5 dicaffeoylquinic acid	hydroxycinnamates
6.27	581.1866	461 (100), 491 (26), 299 (13)	C_27_H_33_O_14_	11.5	−1.719	kaempferid pentoside hexoside	flavonol glycosides
6.44	355.1396	173 (100), 161 (22), 143 (21)	C_17_H_23_O_8_	6.5	−0.538	9-*O*-methylconiferin	phenylpropanoids
7.42	417.1183	211 (100), 237 (25), 255 (13)	C_21_H_21_O_9_	11.5	−1.955	liquiritine	flavanone glycosides
7.57	193.0506	-	C_10_H_9_O_4_	6.5	−0.011	ferulic acid	hydroxycinnamates
8.25	549.1609	255 (100), 429 (26), 297 (21), 279 (8)	C_26_H_29_O_13_	12.5	−0.863	pinocembrin arabinosyl glucoside	flavanone glycosides
8.50	563.1763	255 (100), 297 (27), 443 (26), 401 (8)	C_27_H_31_O_13_	12.5	−1.321	pinocembrin neohesperidoside	flavanone glycosides
8.63	591.1711	255 (100), 549 (57), 429 (42), 279 (18), 297 (15)	C_28_H_31_O_14_	13.5	−1.436	pinocembrin acetyl arabinosyl glucoside	flavanone glycosides
9.04	417.1189	255 (100)	C_21_H_21_O_9_	11.5	−0.564	pinocembroside	flavanone glycosides
9.06	605.1867	255 (100), 563 (97), 545 (49), 443 (40), 401 (12)	C_29_H_33_O_14_	13.5	−1.436	pinocembrin acetyl neohesperidoside	flavanone glycosides
9.59	255.0664	213 (100), 211 (42), 151 (34), 187 (17), 145 (12), 169 (10)	C_15_H_11_O_4_	10.5	0.423	pinocembrin	flavanones
9.82	459.1291	-	C_23_H_23_O_10_	12.5	−1.263	pinocembrin acetyl glucoside	flavanone glycosides
10.01	591.1711	255 (100), 429 (50), 549 (20), 279 (17), 297 (16)	C_28_H_31_O_14_	13.5	−1.436	pinocembrin acetyl arabinosyl glucoside	flavanone glycosides
10.27	605.1858	-	C_29_H_33_O_14_	13.5	−2.956	pinocembrin acetyl neohesperidoside	flavanone glycosides
10.89	459.1292	255 (100)	C_23_H_23_O_10_	12.5	−1.067	pinocembrin acetyl glucoside	flavanone glycosides
12.80	255.0661	213 (100), 211 (42), 151 (36), 187 (16), 145 (9), 169 (7)	C_15_H_11_O_4_	10.5	−0.714	pinocembrin isomer	flavanones

^1^ Tentative identification.

**Table 2:** Presence (+) or abscence (−) of different compound classes in the studied chórta-enriched decoctions and the corresponding half maximal inhibitory concentration (IC_50_) values determined via DPPH assay.

Botanical Name	*Centaurea raphanina*	*Carthamus lanatus*	*Cichorium intybus*	*Cichorium endivia*	*Cichorium spinosum*	*Crepis sancta*	*Sonchus asper*	*Amaranthus blitum*
Common Greek Name	Agkinaráki	Gourounáki	Radíki	Agourorádiko	Stamnagkáthi	Ladáki	Zochós	Vlíto
Small dicarboxylic acids	+	+	+	+	+	+	+	+
Caffeoyl-quinic acids	+	+	+	+	+	+	+	−
Caffeoyl-tartaric acids	−	−	+	+	+	+	+	−
Flavonols and Flavones	+	+	+	+	+	+	+	+
Flavanones (pinocembrin derivatives)	+	−	−	−	−	−	−	−
Sesquiterpene lactones	−	−	+	+	+	+	−	−
DPPH * (IC_50_ μg/mL)	120.60 ± 15.10	8.86 ± 0.82	10.64 ± 0.92	7.51 ± 1.20	15.08 ± 62.05	13.43 ± 2.02	13.56 ± 3.19	85.38 ± 7.51

* Values are the mean ± SD of at least two separate triplicate experiments.

## Supplementary Materials

The following are available online. Figure S1: UPLC-ESI(−)-HRMS chromatograms of Greek edible greens’ decoctions: (a) *Centaurea raphanina*, (b) *Cichorium endivia*, (c) *Cichorium intybus*, (d) *Crepis sancta*, (e) *Cichorium spinosum*, (f) *Sonchus asper*, (g) *Carthamus lanatus*, and (h) *Amaranthus blitum*, Figure S2: Relative (%) survival (MTT assay) of C5N and A5 cells incubated with the indicated concentrations (μg/mL) of the enriched decoction of *Cichorium endivia* for 72 h, Figure S3: ^1^Η nuclear magnetic resonance (NMR) spectrum of pinocembrin 7-*O*-glucoside, 600 MHz, solvent DMSO-*d*_6_. Figure S4: HPLC-PDA (High Performance Liquid Chromatography coupled to a Photodiode Array Detector) chromatogram of *Centaurea raphanina* extract. Detection at 280 nm. Table S1: Retention time (Rt), HRMS data, and proposed identification of detected features in *Cichorium endivia* water decoctions by UHPLC-ESI(−)-HRMS, Table S2: Retention time (Rt), HRMS data, and proposed identification of detected features in *Cichorium intybus* water decoctions by UHPLC-ESI(−)-HRMS, Table S3: Retention time (Rt), HRMS data, and proposed identification of detected features in *Crepis sancta* water decoctions by UHPLC-ESI(−)-HRMS, Table S4: Retention time (Rt), HRMS data, and proposed identification of detected features in *Cichorium spinosum* water decoctions by UHPLC-ESI(−)-HRMS, Table S5: Retention time (Rt), HRMS data, and proposed identification of detected features in *Sonchus asper* water decoctions by UHPLC-ESI(−)-HRMS, Table S6: Retention time (Rt), HRMS data, and proposed identification of detected features in *Carthamus lanatus* water decoctions by UHPLC-ESI(−)-HRMS, Table S7: Retention time (Rt), HRMS data, and proposed identification of detected features in *Amaranthus blitum* water decoctions by UHPLC-ESI(−)-HRMS. Table S8: ^1^H, ^13^C spectral data of pinocembrin 7-*O*-glucoside (4), 600 MHz, DMSO-*d*_6_. Table S9: Relative quantification of major secondary metabolites in *Centaurea raphanina*’s decoction at 280 nm.
