# Supplementary material for: Phytochemical Composition of the Decoctions of Greek Edible Greens (Chórta) and Evaluation of Antioxidant and Cytotoxic Properties
Source: Molecules. 2018 Jun 26;23(7):1541. doi: 10.3390/molecules23071541 (PMC6100566; doi:10.3390/molecules23071541)
Supplement: Supplementary file 1 [file molecules-23-01541-s001.pdf]

Article

Phytochemical Composition of the Decoctions of Greek Edible Greens (Chórta) and Evaluation of Antioxidant and Cytotoxic Properties

Eleni V. Mikropoulou [1], Konstantina Vougogiannopoulou [1], Eleftherios Kalpoutzakis [1], Aimilia D. Sklirou [2], Zoi Skaperda [3], Joëlle Houriet [4], Jean-Luc Wolfender [4], Ioannis P. Trougakos [2], Dimitrios Kouretas [3], Maria Halabalaki [1,*] and Sofia Mitakou [1]

[1] Department of Pharmacognosy and Natural Products Chemistry, Faculty of Pharmacy, National and Kapodistrian University of Athens, Panepistimiopolis Zografou, 15771 Athens, Greece; elenamik@pharm.uoa.gr (E.V.M.); nadia_voug@pharm.uoa.gr (K.V.); elkalp@pharm.uoa.gr (E.K.); mitakou@pharm.uoa.gr (S.M.)

[2] Department of Cell Biology and Biophysics, Faculty of Biology, National and Kapodistrian University of Athens, Panepistimiopolis Zografou, 15784 Athens, Greece; asklirou@biol.uoa.gr (A.D.S.); itrougakos@biol.uoa.gr (I.P.T.)

[3] Department of Biochemistry and Biotechnology, University of Thessaly, 41221 Larissa, Greece; zoskaper@bio.uth.gr (Z.S.); dkouret@uth.gr (D.K.)

[4] Phytochemistry and Bioactive Natural Products, School of Pharmaceutical Sciences, University of Geneva, University of Lausanne, CMU—Rue Michel-Servet 1, 1206 Geneva, Switzerland; Joelle.Houriet@unige.ch (J.H.); Jean-Luc.Wolfender@unige.ch (J.-L.W.);

* Correspondence: mariahal@pharm.uoa.gr; Tel.: +30-210-727-4781

Table of Contents

Figure S1. UPLC-ESI(-)-HRMS full scan chromatograms of Greek edible greens' decoctions: (a) *Centaurea raphanina,* (b) *Cichorium endivia,* (c) *Cichorium intybus,* (d) *Crepis sancta,* (e) *Cichorium spinosum,* (f) *Sonchus asper,* (g) *Carthamus lanatus,* (h) *Amaranthus blitum.* 1

Table S1. Retention time (Rt), HRMS data, and proposed identification of detected features in *Cichorium endivia* water decoctions by UHPLC-ESI(-)-HRMS. 2

Table S2. Retention time (Rt), HRMS data, and proposed identification of detected features in *Cichorium intybus* water decoctions by UHPLC-ESI(-)-HRMS. 3

Table S3. Retention time (Rt), HRMS data, and proposed identification of detected features in *Crepis sancta* water decoctions by UHPLC-ESI(-)-HRMS. 4

Table S4. Retention time (Rt), HRMS data, and proposed identification of detected features in *Cichorium spinosum* water decoctions by UHPLC-ESI(-)-HRMS. 5

Table S5. Retention time (Rt), HRMS data, and proposed identification of detected features in *Sonchus asper* water decoctions by UHPLC-ESI(-)-HRMS. 7

Table S6. Retention time (Rt), HRMS data, and proposed identification of detected features in *Carthamus lanatus* water decoctions by UHPLC-ESI(-)-HRMS. 8

Table S7. Retention time (Rt), HRMS data, and proposed identification of detected features in *Amaranthus blitum* water decoctions by UHPLC-ESI(-)-HRMS. 9

Figure S2. Relative (%) survival (MTT assay) of C5N and A5 cells incubated with the indicated concentrations (μg/ml) of the enriched decoction of *Cichorium endivia* for 72 h. 10

Figure S3. ^{1}H NMR spectrum of pinocembrin 7-O-glucoside, 600 MHz, solvent DMSO-d_6. 11

Table S8. ^{1}H, ^{13}C spectral data of pinocembrin 7-*O*-glucoside (4), 600 MHz, DMSO-d_6. 12

Figure S4. HPLC-PDA chromatogram of *Centaurea raphanina* extract. Detection at 280 nm. 13

Table S9. Relative quantification of major secondary metabolites in *Centaurea raphanina's* decoction at 280 nm. 13

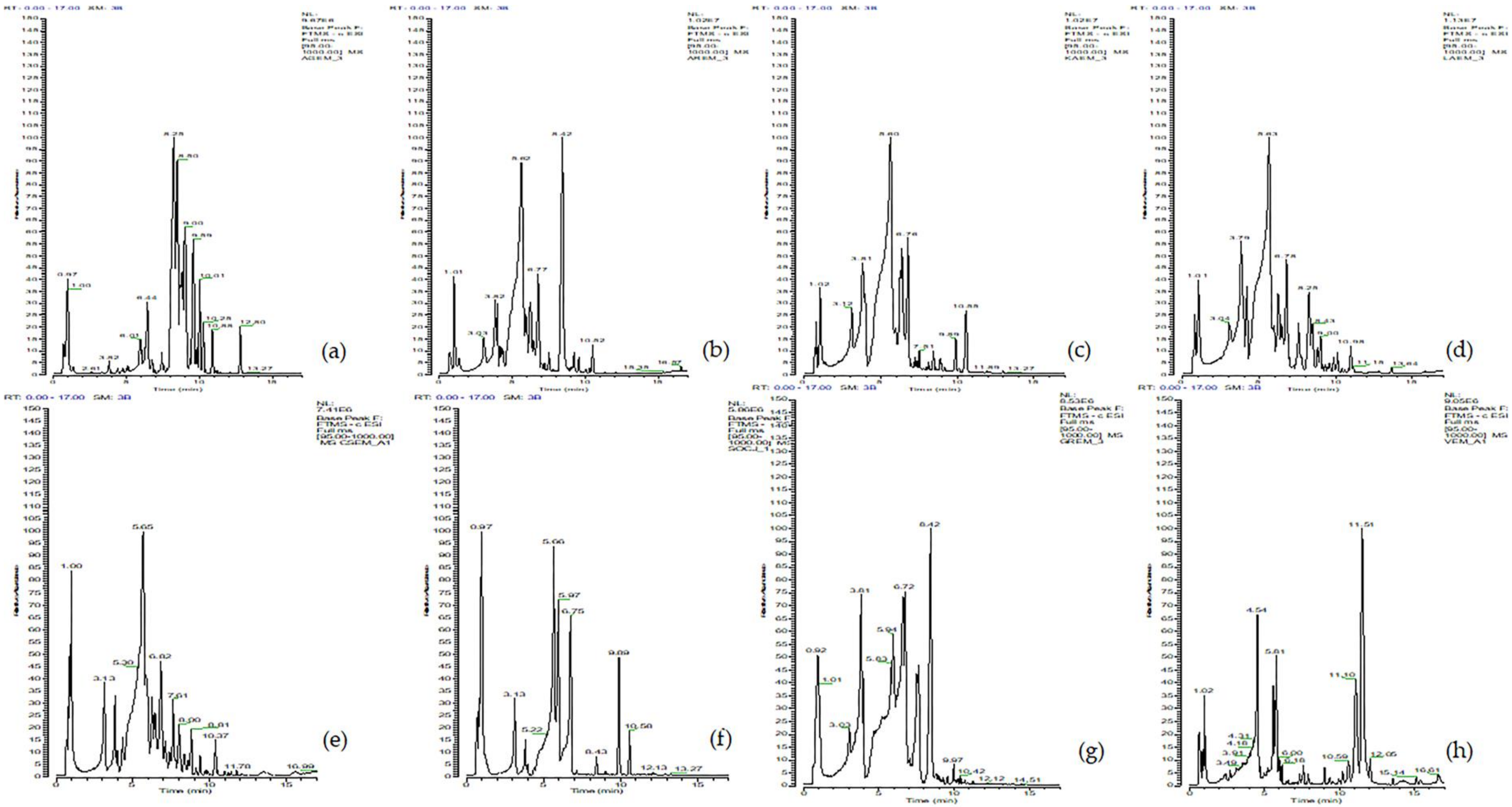

Figure S1. UPLC-ESI(-)-HRMS full scan chromatograms of Greek edible greens' decoctions: (**a**) *Centaurea raphanina,* (**b**) *Cichorium endivia,* (**c**) *Cichorium intybus,* (**d**) *Crepis sancta,* (**e**) *Cichorium spinosum,* (**f**) *Sonchus asper,* (**g**) *Carthamus lanatus,* (**h**) *Amaranthus blitum.*

Table S1. Retention time (Rt), HRMS data, and proposed identification of detected features in *Cichorium endivia* water decoctions by UHPLC-ESI(-)-HRMS.

Retention Time (min)	Detected *m/z* ([M-H]-)	HRMS/MS fragment ions (relative intensity)	Elemental Composition	RDBeq.	*Δ* (ppm)	Compound[1]	Chemical Class
0.76	146.0463	-	$C_5H_8O_4N$	2.5	3.143	glutamic acid	organic acids
0.91	133.0146	-	$C_4H_5O_5$	2.5	0.179	malic acid	organic acids
0.98	191.0198	-	$C_6H_7O_7$	3.5	0.179	citric cid	organic acids
1.06	117.0199	-	$C_4H_5O_4$	2.5	4.598	succinic acid	organic acids
3.03	353.0873	191 (100), 179 (35), 173 (26), 135 (5)	$C_{16}H_{17}O_9$	8.5	-1.488	3-caffeoylquinic acid	hydroxycinnamates
3.13	311.0404	149 (100), 179 (58)	$C_{13}H_{11}O_9$	8.5	-1.335	caftaric acid	hydroxycinnamates
3.83	353.0878	191 (100), 179 (10), 173 (10), 135 (1)	$C_{16}H_{17}O_9$	8.5	-0.808	5-caffeoylquinic acid	hydroxycinnamates
3.99	353.0874	-	$C_{16}H_{17}O_9$	8.5	-1.063	caffeoylquinic acid isomer	hydroxycinnamates
4.28	609.1445	285 (100)	$C_{27}H_{29}O_{16}$	13.5	-2.590	luteolin diglycoside	flavonol glycosides
4.34	177.0195	133 (100), 149 (8)	$C_9H_5O_4$	7.5	1.232	esculetin	coumarins
5.62	473.0729	311 (100), 293 (57)	$C_{22}H_{17}O_{12}$	14.5	-0.273	cichoric acid	hydroxycinnamates
5.88	447.0932	285 (100)	$C_{21}H_{19}O_{11}$	12.5	-0.256	luteolin glucoside	flavonol glycosides
5.91	461.0722	285 (100)	$C_{21}H_{17}O_{12}$	13.5	-0.670	luteolin glucuronide	flavonol glucuronides
6.17	433.0771	301 (100)	$C_{20}H_{17}O_{11}$	12.5	-1.119	quercetin pentoside	flavone glycosides
6.27	515.1191	353 (100), 335 (8), 173 (6), 179 (4), 191 (3)	$C_{25}H_{23}O_{12}$	14.5	-0.697	3,4-dicaffeoylquinic acid	hydroxycinnamates
6.43	341.0696	-	$C_{15}H_{17}O_7S$	7.5	-1.369	deacetylmatricarin-8-*O*-sulfate	sesquiterpene lactones
6.47	457.0774	295 (100), 293 (44), 277 (34)	$C_{22}H_{17}O_{11}$	14.5	-0.535	*p*-coumaroylcaffeoyltartaric acid	hydroxycinnamates
6.78	515.1191	353 (100), 299 (10), 335 (4)	$C_{25}H_{23}O_{12}$	14.5	-0.697	3,5-dicaffeoylquinic acid	hydroxycinnamates
6.88	487.0873	325 (100), 293 (54), 307 (35)	$C_{23}H_{19}O_{12}$	14.5	-1.887	feruloylcaffeoyltartaric acid	hydroxycinnamates
7.02	447.0926	285 (100)	$C_{21}H_{19}O_{11}$	12.5	-1.621	kaempferol glucoside	flavonol glycosides
7.23	261.1129	217 (100)	$C_{15}H_{17}O_4$	7.5	-1.196	helenalin	sesquiterpene lactones
8.44	285.0406	-	$C_{15}H_9O_6$	11.5	0.452	luteolin	flavonols
9.25	421.0923	311 (100)	$C_{23}H_{17}O_8$	15.5	-1.522	unknown	-
9.57	269.0452	-	$C_{15}H_9O_5$	11.5	-1.289	apigenin	flavones

[1] Tentative Identification

Table S2. Retention time (Rt), HRMS data, and proposed identification of detected features in *Cichorium intybus* water decoctions by UHPLC-ESI(-)-HRMS.

Retention Time (min)	Detected *m/z* ([M-H]-)	HRMS/MS fragment ions (relative intensity)	Elemental Composition	RDBeq.	*Δ* (ppm)	Compound[2]	Chemical Class
0.74	146.0462	-	$C_5H_8O_4N$	2.5	1.910	glutamic acid	organic acids
0.75	225.0615	-	$C_7H_{13}O_8$	1.5	-0.447	heptonic acid	organic acids
0.78	195.0511	-	$C_6H_{11}O_7$	1.5	0.482	gluconic acid	organic acids
0.80	191.0562	111 (100)	$C_7H_{11}O_6$	2.5	0.464	quinic acid	organic acids
0.82	165.0407	-	$C_5H_9O_6$	1.5	1.689	pentonic acid	organic acids
0.84	149.0094	-	$C_4H_5O_6$	2.5	1.603	tartaric acid	organic acids
0.91	133.0146	-	$C_4H_5O_5$	2.5	2.357	malic acid	organic acids
1.03	117.0199	-	$C_4H_5O_4$	2.5	4.598	succinic acid	organic acids
3.02	353.0869	191 (100). 179 (32), 173 (11), 135 (5)	$C_{16}H_{17}O_9$	8.5	-2.536	3-caffeoylquinic acid	hydroxycinnamates
3.12	311.0403	149 (100), 179 (59)	$C_{13}H_{11}O_9$	8.5	-1.946	caftaric acid	hydroxycinnamates
3.38	339.0712	177 (100)	$C_{15}H_{15}O_9$	8.5	-2.729	cichoriin	coumarins
3.81	353.0873	191 (100), 173 (13), 179 (11), 135 (1)	$C_{16}H_{17}O_9$	8.5	-1.403	5-caffeoylquinic acid	hydroxycinnamates
4.28	595.1287	301 (100)	$C_{26}H_{27}O_{16}$	13.5	-3.021	quercetin pentoside hexoside	flavone glycosides
4.32	609.1445	285 (100), 447 (9)	$C_{27}H_{29}O_{16}$	13.5	-2.689	luteolin diglycoside	flavonol glycosides
5.64	473.0714	311 (100), 293 (68)	$C_{22}H_{17}O_{12}$	14.5	-2.535	cichoric acid	hydroxycinnamates
5.89	447.0927	285 (100)	$C_{21}H_{19}O_{11}$	12.5	-1.419	luteolin glucoside	flavonol glycosides
5.91	461.0717	285 (100)	$C_{21}H_{17}O_{12}$	13.5	-1.863	luteolin glucuronide	flavonol glucuronides
6.25	515.1190	353 (100), 335 (8), 173 (6), 179 (4), 191 (2)	$C_{25}H_{23}O_{12}$	14.5	-1.047	3,4-dicaffeoylquinic acid	hydroxycinnamates
6.41	341.0696	97 (100)	$C_{15}H_{17}O_7S$	7.5	-1.193	deacetylmatricarin-8-*O*-sulfate	sesquiterpene lactones
6.53	457.0769	295 (100), 293 (44), 277 (39)	$C_{22}H_{17}O_{11}$	14.5	-1.673	*p*-coumaroylcaffeoyltartaric acid	hydroxycinnamates
6.76	445.0772	269 (100)	$C_{21}H_{17}O_{11}$	13.5	-0.954	apigenin glucuronide	flavone glucuronides
6.78	515.1191	353 (100), 299 (10), 203 (8), 335 (3)	$C_{25}H_{23}O_{12}$	14.5	-0.814	3,5-dicaffeoylquinic acid	hydroxycinnamates
7.22	261.1129	217 (100)	C15H17O4	7.5	-1.081	helenalin	sesquiterpene lactones
7.31	441.0818		$C_{22}H_{17}O_{10}$	14.5	-2.108	di-*p*-coumaroyltartaric acic	hydroxycinnamates
8.43	285.0399	-	$C_{15}H_9O_6$	11.5	-1.899	luteolin	flavonols
9.87	601.1735	-	$C_{33}H_{29}O_{11}$	19.5	3.003	unknown	-
10.55	327.0907	97 (100)	$C_{15}H_{19}O_6S$	6.5	-1.658	unknown	sesquiterpene lactones

[2] Tentative Identification

Table S3. Retention time (Rt), HRMS data, and proposed identification of detected features in Crepis sancta water decoctions by UHPLC-ESI(-)-HRMS.

Retention Time (min)	Detected m/z ($[M-H]^-$)	HRMS/MS fragment ions (relative intensity)	Elemental Composition	RDBeq.	Δ (ppm)	Compound[3]	Chemical Class
0.76	165.0409	-	$C_5H_9O_6$	1.5	2.537	pentonic acid	organic acids
0.78	195.0511	-	$C_6H_{11}O_7$	1.5	0.328	gluconic acid	organic acids
0.82	149.0096	-	$C_4H_5O_6$	2.5	3.012	tartaric acid	organic acids
0.84	191.0563	173 (100)	$C_7H_{11}O_6$	2.5	0.778	quinic acid	organic acids
0.91	133.0147	-	$C_4H_5O_5$	2.5	3.484	malic acid	organic acids
1.01	191.0201	-	$C_6H_7O_7$	3.5	2.011	citric acid	organic acids
3.05	353.0874	191 (100), 179 (20), 173 (11), 135 (3)	$C_{16}H_{17}O_9$	8.5	-1.233	3-caffeoylquinic acid	hydroxycinnamates
3.13	311.0405	149 (100), 179 (58)	$C_{13}H_{11}O_9$	8.5	-1.238	caftaric acid	hydroxycinnamates
3.37	339.0717	177 (100)	$C_{15}H_{15}O_9$	8.5	-1.372	cichoriin	coumarins
3.83	353.0877	191 (100), 179 (8), 173 (8)	$C_{16}H_{17}O_9$	8.5	-0.185	5-caffeoylquinic acid	hydroxycinnamates
3.97	353.0876	-	$C_{16}H_{17}O_9$	8.5	-0.610	caffeoylquinic acid isomer	hydroxycinnamates
4.29	595.1293	463 (100), 433 (78), 301 (5)	$C_{26}H_{27}O_{16}$	13.5	-0.710	quercetin pentoside hexoside	flavone glycosides
4.31	609.1448	285 (100), 447 (22)	$C_{27}H_{29}O_{16}$	13.5	0.601	luteolin diglycoside	flavonol glycosides
5.63	473.0722	311 (100), 293 (61)	$C_{22}H_{17}O_{12}$	14.5	-0.801	cichoric acid	hydroxycinnamates
5.94	447.0931	285 (100)	$C_{21}H_{19}O_{11}$	12.5	-0.390	luteolin glucoside	flavonol glycosides
5.98	461.0721	285 (100)	$C_{21}H_{17}O_{12}$	13.5	-1.017	luteolin glucuronide	flavonol glucuronides
6.20	433.0775	301 (100)	$C_{20}H_{17}O_{11}$	12.5	-0.426	quercetin pentoside	flavone glycosides
6.26	515.1193	353 (100), 335 (7), 299 (2)	$C_{25}H_{23}O_{12}$	14.5	-0.465	3,4-dicaffeoylquinic acid	hydroxycinnamates
6.63	431.0979	269 (100)	$C_{21}H_{19}O_{10}$	12.5	-1.160	apigenin glucoside	flavone glycosides
6.78	515.1191	353 (100), 299 (9), 335 (4)	$C_{25}H_{23}O_{12}$	14.5	-0.697	3,5-dicaffeoylquinic acid	hydroxycinnamates
7.01	447.0924	285 (100)	$C_{21}H_{19}O_{11}$	12.5	1.631	kaempferol glucoside	flavonol glycosides
7.20	261.1130	217 (100)	$C_{15}H_{17}O_4$	7.5	-0.966	helenalin	sesquiterpene lactones
8.43	285.0403	-	$C_{15}H_9O_6$	11.5	-0.636	luteolin	flavonols
8.50	301.0351	-	$C_{15}H_9O_7$	11.5	-0.916	quercetin	flavones
10.14	601.1731	-	$C_{33}H_{29}O_{11}$	19.5	-0.286	unknown	-
10.99	601.1734	-	$C_{33}H_{29}O_{11}$	19.5	0.213	unknown	-

[3] Tentative Identification

Table S4. Retention time (Rt), HRMS data, and proposed identification of detected features in *Cichorium spinosum* water decoctions by UHPLC-ESI(-)-HRMS.

Retention Time (min)	Detected *m/z* ([M-H]-)	HRMS/MS fragment ions (relative intensity)	Elemental Composition	RDBeq.	*Δ* (ppm)	Compound[4]	Chemical Class
0.76	165.0408	-	$C_5H_9O_6$	1.5	1.871	pentonic acid	organic acids
0.77	195.0511	-	$C_6H_{11}O_7$	1.5	0.636	gluconic acid	organic acids
0.82	149.0096	-	$C_4H_5O_6$	2.5	2.744	tartaric acid	organic acids
0.84	191.0563	-	$C_7H_{11}O_6$	2.5	1.197	quinic acid	organic acids
0.91	133.0147	-	$C_4H_5O_5$	2.5	3.108	malic acid	organic acids
1.00	191.0200	-	$C_6H_7O_7$	3.5	1.278	citric acid	organic acids
1.22	225.0614	-	$C_7H_{13}O_8$	1.5	-1.025	heptonic acid	organic acids
3.06	353.0870	191 (100), 179 (15), 173 (9)	$C_{16}H_{17}O_9$	8.5	0.271	3-caffeoylquinic acid	hydroxycinnamates
3.14	311.0403	149 (100), 179 (56)	$C_{13}H_{11}O_9$	8.5	-1.656	caftaric acid	hydroxycinnamates
3.41	339.0715	177 (100)	$C_{15}H_{15}O_9$	8.5	0.412	cichoriin	coumarins
3.82	353.0872	191 (100), 173 (9), 179 (7)	$C_{16}H_{17}O_9$	8.5	-1.658	5-caffeoylquinic acid	hydroxycinnamates
3.94	295.0453	163 (100), 149 (27)	$C_{13}H_{11}O_8$	8.5	0.506	coutaric acid	hydroxycinnamates
4.33	325.0559	193 (100), 149 (7)	$C_{14}H_{13}O_9$	8.5	0.502	fertaric acid	hydroxycinnamates
5.06	367.1028	191 (100)	$C_{17}H_{19}O_9$	8.5	0.391	feruloylquinic acid	hydroxycinnamates
5.65	473.0723	311 (100)	$C_{22}H_{17}O_{12}$	14.5	-0.590	cichoric acid	hydroxycinnamates
5.85	477.0670	301 (100)	$C_{21}H_{17}O_{13}$	13.5	-1.056	quercetin glucuronide	flavone glycosides
5.94	461.0722	285 (100)	$C_{21}H_{17}O_{12}$	13.5	-0.670	luteolin glucuronide	flavonol glycosides
6.25	515.1188	353 (100), 335 (10), 179 (4), 191 (2)	$C_{25}H_{23}O_{12}$	14.5	-1.280	3,4-dicaffeoylquinic acid	hydroxycinnamates
6.43	341.0695	97 (100)	$C_{15}H_{17}O_7S$	7.5	-1.457	deacetylmatricarin-8-*O*-sulfate	sesquiterpene lactones
6.75	445.0771	269 (100)	$C_{21}H_{17}O_{11}$	13.5	0.582	apigenin glucuronide	flavone glycosides
6.82	515.1188	353 (100), 299 (9)	$C_{25}H_{23}O_{12}$	14.5	0.438	3,5-dicaffeoylquinic acid	hydroxycinnamates
7.11	487.0872	325 (100), 293 (57), 307 (34), 179 (6)	$C_{23}H_{19}O_{12}$	14.5	-2.010	feruloylcaffeoyltartaric acid	hydroxycinnamates
7.37	677.2808	617 (100), 659 (65), 645 (22)	$C_{34}H_{45}O_{14}$	12.5	-1.062	dimeric sesquiterpene lactone (picrioside type)	sesquiterpene lactones
7.61	677.2806	617 (100), 645 (96), 659 (56), 520 (18)	$C_{34}H_{45}O_{14}$	12.5	-1.239	dimeric sesquiterpene lactone (picrioside type)	sesquiterpene lactones
7.75	441.0819	277 (100), 295 (11)	$C_{22}H_{17}O_{10}$	14.5	-1.904	di-*p*-coymaroyltartaric acid	hydroxycinnamates
7.77	471.0924	307 (100), 277 (84)	$C_{23}H_{19}O_{11}$	14.5	-1.920	coumaroylferuloyltartaric acid	hydroxycinnamates
8.00	501.1032	307 (100)	$C_{24}H_{21}O_{12}$	14.5	-1.216	*p*-coumaroylsinapoyl tartaric acid isomer	hydroxycinnamates
8.38	501.1030	307 (100)	$C_{24}H_{21}O_{12}$	14.5	-1.695	*p*-coumaroylsinapoyl tartaric acid isomer	hydroxycinnamates

[4] Tentative Identification

8.81	805.3268	773 (100)	$C_{40}H_{53}O_{17}$	14.5	-2.537	dimeric sesquiterpene lactone (picrioside type)	sesquiterpene lactones
9.38	409.1285	-	$C_{23}H_{21}O_7$	12.5	-1.800	lactupicrin	sesquiterpene lactones
10.37	643.2753	611 (100), 520 (15), 567 (12)	$C_{34}H_{43}O_{12}$	13.5	-1.135	dimeric sesquiterpene lactone	sesquiterpene lactones

Table S5. Retention time (Rt), HRMS data, and proposed identification of detected features in <u>*Sonchus asper*</u> water decoctions by UHPLC-ESI(-)-HRMS.

**Retention Time (min)**	**Detected *m/z* ($[M-H]^-$)**	**HRMS/MS fragment ions (relative intensity)**	**Elemental Composition**	**RDBeq.**	***Δ* (ppm)**	**Compound[5]**	**Chemical Class**
0.76	195.0511	-	$C_6H_{11}O_7$	1.5	0.380	gluconic acid	organic acids
0.79	165.0408	-	$C_5H_9O_6$	1.5	1.871	heptonic acid	organic acids
0.83	149.0095	-	$C_4H_5O_6$	2.5	2.207	tartaric acid	organic acids
0.84	191.0562	173 (100)	$C_7H_{11}O_6$	2.5	0.516	quinic acid	organic acids
0.90	133.0147	-	$C_4H_5O_5$	2.5	3.334	malic acid	organic acids
1.00	191.0200	111 (100)	$C_6H_7O_7$	3.5	1.592	citric acid	organic acids
1.18	128.0357	-	$C_5H_6O_3N$	3.5	2.684	pyroglutamic acid	organic acids
1.25	135.0301	89 (100)	$C_4H_7O_5$	1.5	1.654	threonic acid	organic acids
3.13	311.0402	149 (100), 179 (60)	$C_{13}H_{11}O_9$	8.5	-2.042	caftaric acid	hydroxycinnamates
3.82	353.0869	191 (100), 173 (12), 179 (9)	$C_{16}H_{17}O_9$	8.5	-2.451	5-caffeoylquinic acid	hydroxycinnamates
5.66	473.0722	311 (100), 293 (62)	$C_{22}H_{17}O_{12}$	14.5	-0.738	cichoric acid	hydroxycinnamates
5.95	461.0720	285 (100)	$C_{21}H_{17}O_{12}$	13.5	-1.213	luteolin glucuronide	flavonol glucuronides
6.19	515.1181	353 (100), 335 (40), 179 (15)	$C_{25}H_{23}O_{12}$	14.5	-2.716	3,4-dicaffeoylquinic acid	hydroxycinnamates
6.75	445.0771	269 (100)	$C_{21}H_{17}O_{11}$	13.5	-1.291	apigenin glucuronide	flavone glucuronides
8.43	285.0399	-	$C_{15}H_9O_6$	11.5	-1.899	luteolin	flavonols
9.89	327.2170	229 (100), 211 (50), 291 (46)	$C_{18}H_{31}O_5$	3.5	-2.101	TriHODE	fatty acids
10.58	329.2326	229 (100), 211 (73)	$C_{18}H_{33}O_5$	2.5	-2.270	TriHOME	fatty acids

[5] Tentative Identification

Table S6. Retention time (Rt), HRMS data, and proposed identification of detected features in *Carthamus lanatus* water decoctions by UHPLC-ESI(-)-HRMS.

Retention Time (min)	Detected *m/z* ([M-H]⁻)	HRMS/MS fragment ions (relative intensity)	Elemental Composition	RDBeq.	*Δ* (ppm)	Compound[6]	Chemical Class
0.78	165.0408	-	$C_5H_9O_6$	1.5	2.355	heptonic acid	organic acids
0.79	377.0856	-	$C_{12}H_{22}O_{11}Cl$	1.5	-0.033	disaccharide chloride	sugars
0.84	191.0562	173 (100)	$C_7H_{11}O_6$	2.5	0.516	quinic acid	organic acids
0.92	133.0147	115 (100)	$C_4H_5O_5$	2.5	3.108	malic acid	organic acids
1.04	191.0200	-	$C_6H_7O_7$	3.5	1.435	citric acid	organic acids
3.05	353.0870	191 (100), 179 (15), 173 (10)	$C_{16}H_{17}O_9$	8.5	-2.252	3-caffeoylquinic acid	hydroxycinnamates
3.81	353.0878	191 (100), 173 (10), 179 (9)	$C_{16}H_{17}O_9$	8.5	-0.440	5-caffeoylquinic acid	hydroxycinnamates
3.98	353.0872	-	$C_{16}H_{17}O_9$	8.5	-1.573	caffeoylquinic acid isomer	hydroxycinnamates
4.64	337.0925	191 (100), 173 (95), 163 (11)	$C_{16}H_{17}O_8$	8.5	-1.248	5-*p*-coumaroylquinic acid	hydroxycinnamates
5.82	463.0875	301 (100)	$C_{21}H_{19}O_{12}$	12.5	-1.467	quercetin glucoside	flavone glycosides
5.94	447.0930	285 (100)	$C_{21}H_{19}O_{11}$	12.5	-0.726	luteolin 7-*O*-glucoside	flavonol glycosides
6.29	515.1190	353 (100), 335 (8)	$C_{25}H_{23}O_{12}$	14.5	-0.930	3,4-dicaffeoylquinic acid	hydroxycinnamates
6.59	505.0984	301 (100)	$C_{23}H_{21}O_{13}$	13.5	-0.800	quercetin acetyl hexose	flavone glycosides
6.72	489.1033	285 (100)	$C_{23}H_{21}O_{12}$	13.5	-1.184	luteolin acetyl glucoside	flavonol glycosides
6.81	515.1187	353 (100), 299 (7)	$C_{25}H_{23}O_{12}$	14.5	-1.649	3,5-dicaffeoylquinic acid	hydroxycinnamates
7.03	447.0925	285 (100)	$C_{21}H_{19}O_{11}$	12.5	-1.688	kaempferol glucoside	flavonol glycosides
7.51	505.0984	301 (100)	$C_{23}H_{21}O_{13}$	13.5	-0.621	quercetin acetyl hexose	flavone glycosides
7.64	489.1042	285 (100)	$C_{23}H_{21}O_{12}$	13.5	-0.550	kaempferol acetyl glucoside	flavonol glycosides
8.43	285.0404	-	$C_{15}H_9O_6$	11.5	-0.320	luteolin	flavonols
9.56	269.0451	-	$C_{15}H_9O_5$	11.5	-1.772	apigenin	flavones

[6] Tentative Identification

Table S7. Retention time (Rt), HRMS data, and proposed identification of detected features in *Amaranthus blitum* water decoctions by UHPLC-ESI(-)-HRMS.

Retention Time (min)	Detected *m/z* ([M-H]-)	HRMS/MS fragment ions (relative intensity)	Elemental Composition	RDBeq.	*Δ* (ppm)	Compound[7]	Chemical Class
0.76	132.0307	-	$C_4H_6O_4N$	2.5	3.780	aspartic acid	organic acids
0.77	146.0463	-	$C_5H_8O_4N$	2.5	2.732	glutamic acid	organic acids
0.79	165.0408	-	$C_5H_9O_6$	1.5	2.234	pentonic acid	organic acids
0.84	209.0304	191 (100)	C6H9O8	2.5	0.955	glucaric acid	organic acids
0.89	133.0147	-	$C_4H_5O_5$	2.5	3.259	malic acid	organic acids
0.98	191.0198	111 (100)	$C_6H_7O_7$	3.5	0.336	citric acid	organic acids
1.03	371.0619	209 (100)	$C_{15}H_{15}O_{11}$	8.5	-0.335	caffeoylglucaric acid	phenolic acids
4.54	353.0511	173 (100),191 (73), 111 (20)	$C_{15}H_{13}O_{10}$	9.5	-0.764	coumaroylhydroxycitric acid	hydroxycinnamates
5.61	609.1448	301 (100)	$C_{27}H_{29}O_{16}$	13.5	-2.180	rutin	flave glycosides
5.80	367.0666	173 (100), 111 (20)	$C_{16}H_{15}O_{10}$	9.5	-1.226	unknown phenylpropanoid	phenylpropanoids
6.00	367.0663	173 (100)	$C_{16}H_{15}O_{10}$	9.5	-2.152	unknown phenylpropanoid	phenylpropanoids
6.17	593.1497	285 (100)	$C_{27}H_{29}O_{15}$	13.5	-2.484	luteolin diglycoside	flavonol glycosides
7.36	463.2538	417 (100)	$C_{22}H_{39}O_{10}$	3.5	-2.354	unknown	possibly megastigmanes
7.93	503.2488	459 (100)	$C_{24}H_{39}O_{11}$	5.5	-1.878	unknown	possibly megastigmanes
10.59	909.4083	777 (100)	$C_{45}H_{65}O_{19}$	13.5	-4.720	-	-
11.06	955.4507	793 (100), 937 (38), 849 (22)	$C_{47}H_{71}O_{20}$	12.5	-3.912	triterpene saponin	triterpene saponins
11.55	925.4404	793 (100), 631 (17)	$C_{46}H_{69}O_{19}$	12.5	-3.763	triterpene saponin (1 glucuronide + 1 glycoside + aliphatic chain)	triterpene saponins
12.05	921.4445	793 (100)	$C_{47}H_{69}O_{18}$	13.5	-4.795	triterpene saponin	triterpene saponins

[7] Tentative Identification

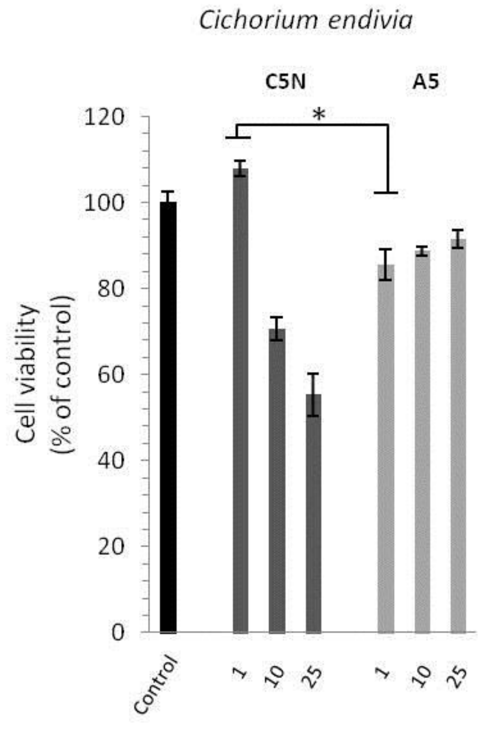

Figure S2. Relative (%) survival (MTT assay) of C5N and A5 cells incubated with the indicated concentrations (μg/ml) of the enriched decoction of *Cichorium endivia* for 72 h.

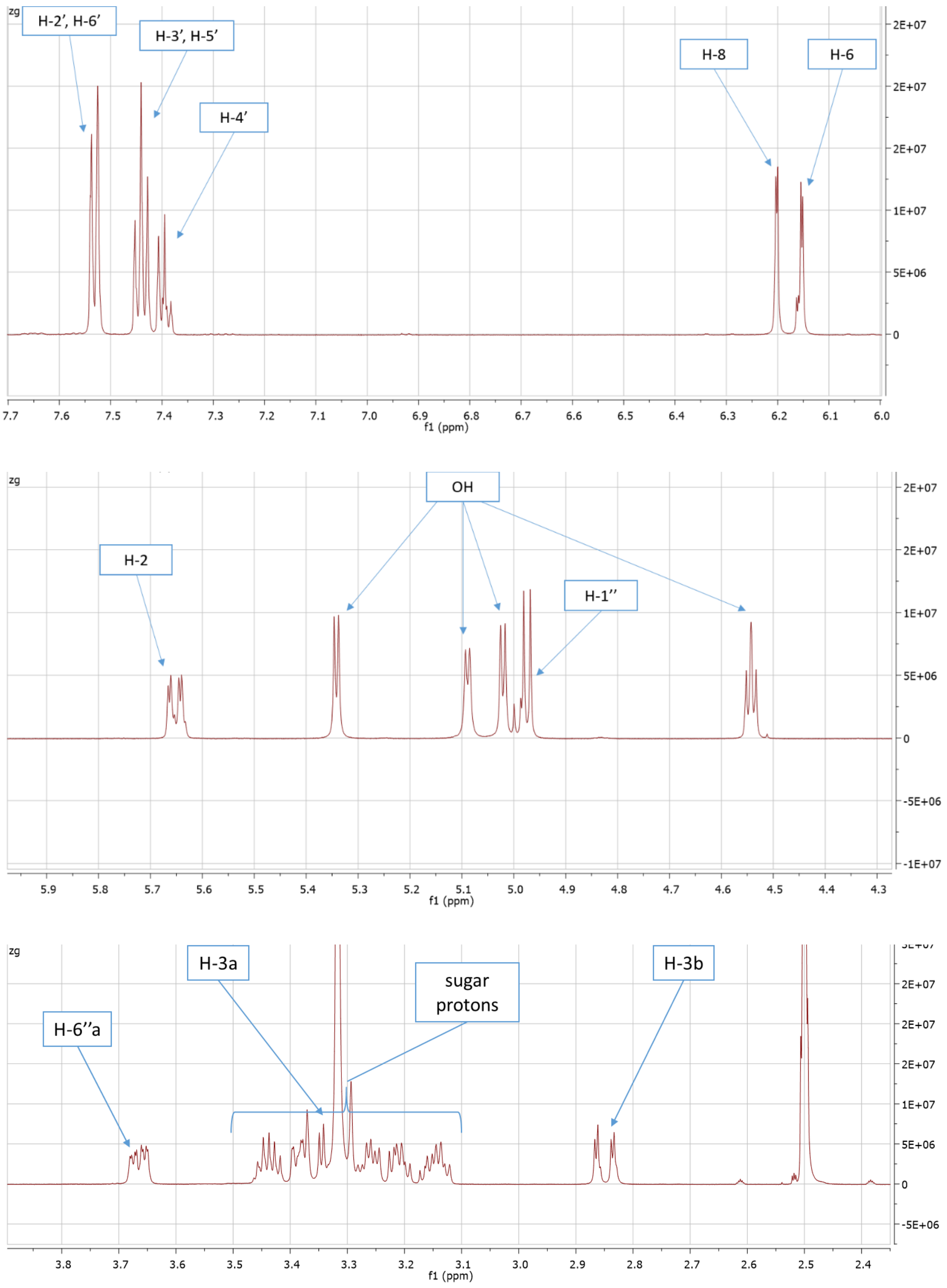

Figure S3. ^{1}H NMR spectrum of pinocembrin 7-*O*-glucoside, 600 MHz, solvent DMSO-d_6.

Table S8. ^{1}H, ^{13}C spectral data of pinocembrin 7-*O*-glucoside (4), 600 MHz, DMSO-d_6.

H	C	^{1}H δ (ppm)	J (Hz)	^{13}C δ (ppm)
2	2	5,65	dd 11,9/2,9	79,0
3a	3	3,35	m	42,5
3b		2,86	dd 17,2/2,9	
-	4	-	-	197,2
-	5	-	-	163,3
6	6	6,15	d 1,8	97,2
-	7	-	-	165,7
8	8	6,20	d 1,8	96,3
-	9	-	-	162,9
-	10	-	-	103,9
-	1′	-	-	139,0
2′	2′	7,53	d 7,6	128,2
3′	3′	7,44	t 7,6	128,0
4′	4′	7,39	t 7,6	129,2
5′	5′	7,44	t 7,6	128,0
6′	6′	7,53	d 7,6	128,2
1′′	1′′	4,97	d 7,7	100,1
2′′	2′′	3,22	m	73,5
3′′	3′′	3,38	m	77,7
4′′	4′′	3,14	m	69,9
5′′	5′′	3,26	m	76,8
6′′a	6′′	3,66	m	60,9
6′′b		3,44	m	
5-OH	-	12,03	brs	-

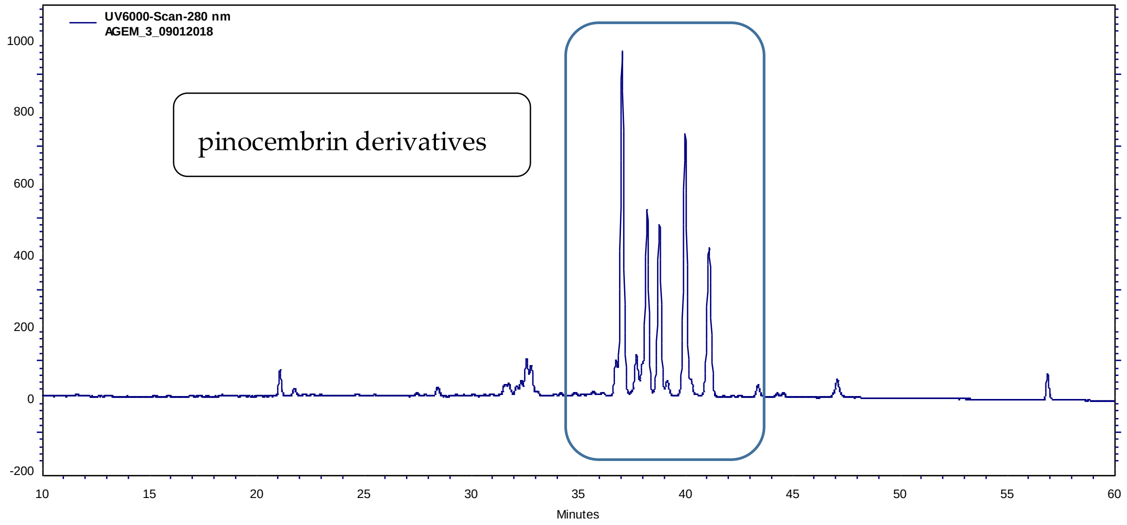

Figure S4. HPLC-PDA chromatogram of *Centaurea raphanina* extract. Detection at 280 nm.

Sample concentration: 1.5 mg/mL in MeOH/ H_2O 50:50. Thermo Finnigan® HPLC-PDA System (P4000 Pump, AS3000 Autosampler, PDA Detector UV8000, ChromquestTM 4.2 Software) and a Supelco® RP18 Discovery HS-C18 (250 mm, 4.6 mm, 5 μm). **Injection volume:** 20 μL. **Mobile phase:** f 0.1% formic acid in water (A) and MeOH (B). **Flow rate:** 1 mL/min**. Column temperature:** 25 °C. **Elution method:** 2% (B), reaching 100% (B) in 60 min and kept for 4 min before getting back to initial conditions in 2 min for a 4-minute re-equilibration.

Table S9. Relative quantification of major secondary metabolites in *Centaurea raphanina's* decoction at 280 nm.

Compound	Area % at 280 nm
pinocembrin arabinosyl glucoside	25.2
pinocembrin neohesperidoside	14.3
pinocembrin acetyl arabinosyl glucoside	12.1
pinocembroside	18.6
pinocembrin	12.4
